# Orthopaedic regenerative tissue engineering en route to the holy grail: disequilibrium between the demand and the supply in the operating room

**DOI:** 10.1186/s40634-018-0133-9

**Published:** 2018-05-22

**Authors:** Ibrahim Fatih Cengiz, Hélder Pereira, Laura de Girolamo, Magali Cucchiarini, João Espregueira-Mendes, Rui L. Reis, Joaquim Miguel Oliveira

**Affiliations:** 1 3B’s Research Group, I3Bs – Research Institute on Biomaterials, Biodegradables and Biomimetics, University of Minho, Headquarters of the European Institute of Excellence on Tissue Engineering and Regenerative Medicine, AvePark, Parque de Ciência e Tecnologia, Zona Industrial da Gandra, 4805-017 Barco, Guimarães, Portugal; 2 ICVS/3B’s – PT Government Associate Laboratory, Braga/Guimarães, Portugal; 3 Ripoll y De Prado Sports Clinic: Murcia-Madrid FIFA Medical Centre of Excellence, Madrid, Spain; 4 Orthopedic Department Centro Hospitalar Póvoa de Varzim, Vila do Conde, Portugal; 5 Orthopaedic Biotechnology Laboratory, IRCCS Galeazzi Orthopaedic Institute, Milan, Italy; 6 Center of Experimental Orthopaedics, Saarland University Medical Center, Kirrbergerstr Bldg 37, D-66421 Homburg/Saar, Germany; 7 Clínica do Dragão, Espregueira-Mendes Sports Centre – FIFA Medical Centre of Excellence, Porto, Portugal; 8 Dom Henrique Research Centre, Porto, Portugal; 9 Orthopedic Department, University of Minho, Braga, Portugal; 10 The Discoveries Centre for Regenerative and Precision Medicine, Headquarters at University of Minho, Avepark, 4805-017 Barco, Guimarães, Portugal

**Keywords:** Clinical, Translation, Scaffold, Stem cells, Bone, Cartilage, Muscle, Ligament, Tendon

## Abstract

Orthopaedic disorders are very frequent, globally found and often partially unresolved despite the substantial advances in science and medicine. Their surgical intervention is multifarious and the most favourable treatment is chosen by the orthopaedic surgeon on a case-by-case basis depending on a number of factors related with the patient and the lesion. Numerous regenerative tissue engineering strategies have been developed and studied extensively in laboratory through in vitro experiments and preclinical in vivo trials with various established animal models, while a small proportion of them reached the operating room. However, based on the available literature, the current strategies have not yet achieved to fully solve the clinical problems. Thus, the gold standards, if existing, remain unchanged in the clinics, notwithstanding the known limitations and drawbacks. Herein, the involvement of regenerative tissue engineering in the clinical orthopaedics is reviewed. The current challenges are indicated and discussed in order to describe the current disequilibrium between the needs and solutions made available in the operating room. Regenerative tissue engineering is a very dynamic field that has a high growth rate and a great openness and ability to incorporate new technologies with passion to edge towards the Holy Grail that is functional tissue regeneration. Thus, the future of clinical solutions making use of regenerative tissue engineering principles for the management of orthopaedic disorders is firmly supported by the clinical need.

## Facing the current challenges

There are numerous challenges that reason the disequilibrium between the clinical demand and the functional supply which are discussed in this review. The challenges and outstanding issues are multifarious and multifactorial. However, from the functional point of view, they fall under the umbrella of the expectation from a typical regenerative tissue engineering product to perform better over time (in both short- and long-term) than the day of implantation. This fact naturally stems from the typical perception of regenerative tissue engineering (i.e. achieving tissue regeneration through matrix synthesis of cells and degradation of scaffold), and reveals why there is a big difference between materials science and biomaterials science on accomplishing their goals. This path to our ultimate goal, our dream, has been very expensive and very time-consuming.

**Fig. 1:**
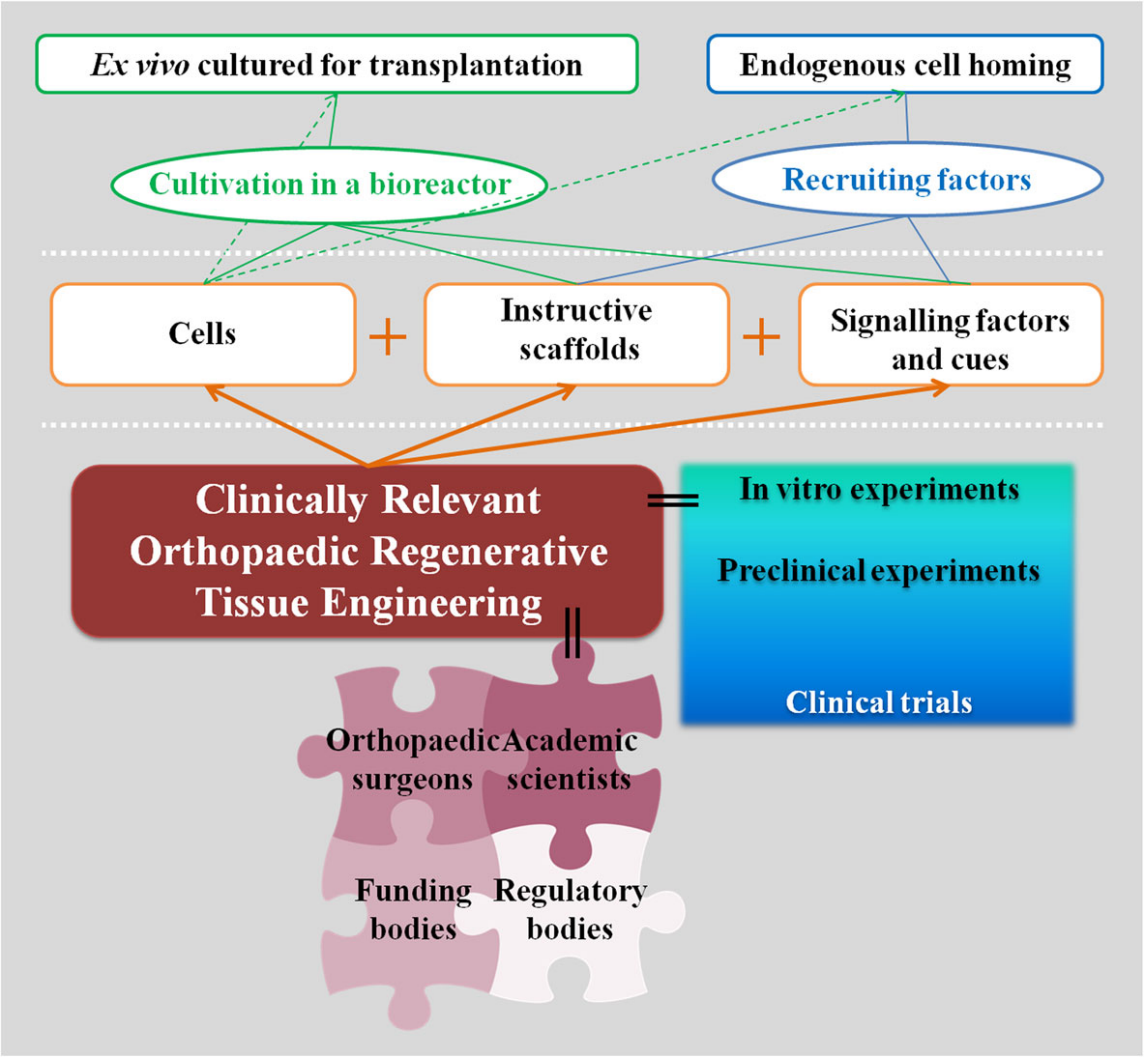
Clinically relevant orthopaedic regenerative tissue engineering strategies. Cells, scaffolds, and signalling factors are the main components of regenerative tissue engineering. Ex vivo cultured cells can be transplanted with or without cultivation in a bioreactor or an in situ tissue regeneration strategy can be followed for endogenous cell homing with the recruited factors such as instructive scaffolds and/or signalling factors can be used. Strong evidence from in vitro and preclinical experiments are needed prior to initiating a clinical trial in humans. Strategies should be developed in a translational research environment with the involvement and communication all stakeholders since the beginning which include orthopaedic surgeons, academic scientists, funding bodies, and regulatory bodies

Typically, regenerative tissue engineering employs cells (Huang et al. 2016), scaffolds (Hollister 2009a; Roffi et al. 2017) or hydrogels (Annabi et al. 2014; Bacelar et al. 2017), and growth/stimulating factors (Gothard et al. 2014; Kwon et al. 2016) while these components have also been used alone for several reasons including relative regulatory, practical and economical convenience. With respect to the involvement of cells in the regenerative tissue engineering, the strategies can comprise the recruitment of the patient’s own cells or the transplantation of cells (Fig. 1). As soon as the cells are in contact with a scaffold, biology “differentiates” into materiobiology, and the behaviour of the cells depend on the features of the scaffold including but not limited to micro-structure, surface properties, and mechanical properties. Besides, the influence of ex vivo culturing of the isolated cells, and the presence of signalling factors affect the cell behaviour. Instructive scaffold strategies promote tissue regeneration with recruitment of scaffold-driven endogenous stem cells that can provide regenerative micro-environments thanks to their paracrine activity (Caplan 2007; Karp and Teo 2009). This strategy comes with the advantages in terms of surgery, cost, regulations, and commercialisation thanks to being an off-the-shelf product (Martin et al. 2007). The typical regenerative tissue engineering strategy involves the scaffolds seeded with ex vivo cultured cell and the cell-scaffold is either maturated in body after implantation assuming the body of the patient as a bioreactor, or cultivated in a bioreactor prior implantation. This strategy lacks the advantages of acellular scaffolds but have a great theoretical potential of functional tissue regeneration. The recruitment of the cells, i.e. endogenous cell homing (Chen et al. 2011; Fong et al. 2011; Karp and Teo 2009; Ko et al. 2013) can be mediated by recruiting factors that are signalling molecules, controlled release of navigational cues in addition to the cues of an instructive scaffold with the rationale of enhancing the intrinsic in situ tissue regeneration, and has been studied in animal models (Burks et al. 2013; Huang et al. 2014; Lee et al. 2008; Shen et al. 2010). Understanding the extremely complex pathways and interactions of the components will bring this strategy a step closer to develop robust clinical treatments.

A regenerative tissue engineering product needs to be extensively studied in vitro (Caddeo et al. 2017), in vivo (McCullen et al. 2011) with animal models (Madry et al. 2015; Moran et al. 2016), and in silico (Díaz-Zuccarini and Lawford 2010; Geris 2014; Geris et al. 2016) whenever possible, before initiating a trial in patients to assure “*Primum non nocere*” (First do no harm) that is present in the Hippocratic oath. Over the last two decades, numerous papers have been published on the tissue engineering of bone (Amini et al. 2012; Oryan et al. 2014), cartilage (Huang et al. 2016; Hunziker et al. 2015), osteochondral tissue (Cengiz et al. 2014; Yan et al. 2015), meniscus (Cengiz et al. 2017b; Cengiz et al. 2017e), tendons (Walden et al. 2017; Youngstrom and Barrett 2015), ligaments (Hogan et al. 2015; Mengsteab et al. 2016), and muscles (Grasman et al. 2015; Sicari et al. 2015). The original works mostly involve in vitro studies, with a certain share of preclinical animal studies. Studies with large animal models are required for the market approval of the product, however, the human body is not the same as any animal model it is clear that no animal model accurately mimics the human condition (Madry et al. 2014).

The ultimate goal of the researcher and of the orthopaedic surgeon in the field is to achieve tissue regeneration to minimise the post-surgery health problems, speed up the patient’s return to activity, and avoid the need of subsequent surgical interventions.

Several distinct tissues are involved in orthopaedics and rigorously require specific considerations since each tissue has its own nature regarding biology and biomechanics alongside its interfaces (Cross et al. 2016; Rao et al. 2016; Tellado et al. 2015) with the adjacent tissues. Therefore, the level of success of tissue regeneration is related to the nature of the tissue (Huey et al. 2012). For instance, although vascularised tissues have a substantial self-healing capacity for minor lesions, for large lesions regenerative tissue engineering tackles the problems regarding hypoxia, vascularisation, and angiogenesis.

To stimulate the cell function and enhance the performance, bioreactors have been studied (Jin et al. 2015; Martin et al. 2009; Ravichandran et al. 2017). Better results can be achieved compared with the static cell culture for instance increased matrix synthesis, mineralisation, expression of specific genes, cell proliferation, and differentiation. Based on the design and application, various bioreactors can be developed proving a range of mechanical, chemical, or electrical stimulation in addition to the features of a static cell culture incubator. However, the diffusion of bioreactors to the clinics are limited probably due to the associated costs and application related issues (Salter et al. 2011). It was estimated that bioreactor-cultured bone grafts would have a cost around 10–15 thousand US dollars, that is comparable to other cellular treatments (Salter et al. 2011).

The cell sources and types can be diverse included but not limited to stem cells (Narayanan et al. 2017) such as adipose or bone marrow-derived stem cells, or primary differentiated cells isolated from a particular tissue, the critical point being to employ cells capable of producing the matrix of that particular tissue. The cells regulate/alter their behaviour depending on the niche that they are in, like in the human body. Thus, the biology in a cell culture flask does not remain the same when the cells are in contact with a scaffold. Cells sense almost everything about the scaffolds, including but not limited to chemistry/composition, nano−/micro−/macro-structure such as porosity, pore size and interconnectivity, surface/volume ratio, topography, degradation profile, and mechanical properties such as stiffness (Li et al. 2017; Zajac and Discher 2008). The surface of a scaffold is of major importance since it is interfacing with the cells where several interactions take place, including fluid and protein adhesion, attachment, adhesion and spreading of cells (Meyer and Wiesmann 2006; Stevens and George 2005). Controlling such features of a scaffold would influence the cell function for instance adhesion, growth, matrix synthesis, differentiation, and alignment. This evidences that a scaffold is not a cell transporting vehicle, but it belongs to the niche of the cells that decode the scaffold’s features (Pennesi et al. 2011). Moreover, all these mentioned features of the scaffold affect the clinical treatment outcome (Cengiz et al. 2017b). Thus, tissue engineering should resolve the unclear cell-scaffold relations and address the scaffold-associated outstanding challenges to ease the translation of tissue-specific cell-scaffolds constructs from laboratory to operating room. Notwithstanding, being a pillar of regenerative tissue engineering, and the need for personalised orthopaedic implants(Cengiz et al. 2016; Cengiz et al. 2017c; Oner et al. 2017), the use of scaffolds can be questioned (Huey et al. 2012) since tissue genesis occurs with self-assembly/organisation via signalling and cell-cell contact without any exogenous scaffold in utero. Self-assembly occurs through the minimisation of free energy via cell-cell interactions and the cells unite into a cohesive structure and act as a scaffold for each other (Athanasiou et al. 2013; Hu and Athanasiou 2006).

Despite our advanced knowledge and expertise (Cengiz et al. 2017b; Fernandez-Yague et al. 2015; Henkel et al. 2013; Hogan et al. 2015; Hunziker et al. 2015; Qazi et al. 2015; Walden et al. 2017; Yan et al. 2015), the current treatments have a range of success and most of them (if not all) are far from “ideal” since they do not systematically provide functional tissue regeneration. Although there is no firm recommendation on the best available regenerative tissue engineering construct strategy, we have been learning from all those studies and trials. Regenerative tissue engineering will be able to solve only a portion of clinical challenges, if it can eventually, given the fact that there are indications/contra-indications for the use of regenerative tissue engineering products. While different regenerative tissue engineering strategies/products are competing with each other, from the clinical perspective, they all compete with the conventional treatments that the surgeon is experienced with and are safer options regarding economics and regulations.

The report from Hollister (Hollister 2009b) clearly illustrates the gap between regenerative tissue engineering research and clinical translation in which the need of a change in research paradigm was highlighted and the difficulty of covering the high costs discussed. It is difficult for a two-step surgical treatment to be favoured over one-step surgical treatment provided similar outcomes at much lower costs (Mollon et al. 2013; Mundi et al. 2016). Bayon et al. (Bayon et al. 2014) reviewed the critical role of partnering in the clinical translation of cell-based advanced therapy medicinal products which was discussed and highlighted in the Tissue Engineering & Regenerative Medicine International Society-Europe Industry Symposium in 2013. Additionally, the report from Madry et al. (Madry et al. 2014) from the “Where Science meets Clinics” symposium in 2013 sponsored by the AO Foundation (https://www.aofoundation.org) conveyed the perspectives of each stakeholder of the clinical translation process of orthopaedic regenerative tissue engineering which include academic scientists, clinicians, industry, and regulatory bodies, and stressed the need for “translational research environment” that is the communication of all stakeholders throughout the project.

The poor translation of regenerative tissue engineering to the clinics has been causing a demand and supply disequilibrium, and the tremendous effort of tissue engineers remain somewhat unappreciated. Apparently, it has been recognised that having a fundamental change in the clinical intervention of orthopaedic disorders is not a short-term task. Besides, it is evident that success in the laboratory is a critical determinant for a human trial. However, the success of laboratory experiments can be maximised, and the outcomes of the research can be enhanced when academic researchers and clinicians work together and make joint efforts throughout the development of the clinical solution including the early stages of idea formation. Since the time of the idea generation and proposal preparation, clinical relevance with defined indications and efficacy measures are needed to create a roadmap for on the envisioned solution. Besides, regulatory requirements (Grieshober et al. 2017; Lysaght et al. 2017) should be known by the research team members from the beginning. For Europe, the Regulation (EC) no 1394/2007 of the European Parliament and of the Council of 13 November 2007 on Advanced Therapy Medicinal Products (ATMPs), which is an optional classification procedure, and amending Directive 2001/83/EC and Regulation (EC) No 726/2004 is available on http://eur-lex.europa.eu/legal-content/EN/TXT/PDF/?uri=CELEX:32007R1394&from=EN. Well-organised preclinical, clinical studies, especially multicenter, randomised clinical trials, although very challenging (Büchler et al. 2011; Ergina et al. 2009; Lyman et al. 2016) are extremely costly by all means. However, this is the robust way to study whether there is an evidence that regenerative tissue engineering solutions can be favoured over comparator treatments for targeted clinical conditions or not.

Thanks to their regenerative potential and regulatory and practical advantage (Chahla et al. 2017), biologics including platelet-rich plasma (PRP), bone marrow aspirate concentrate, hyaluronic acid, and stem cells (Gobbi et al. 2017; Narayanan et al. 2017) have been widely used in orthopaedics (Gobbi et al. 2017; Musahl et al. 2017; Utku et al. 2015) including for bone (Malhotra et al. 2013), cartilage (Nakasa et al. 2017), meniscus (Cengiz et al. 2017a), muscle (Canata et al. 2017), tendons (Canata et al. 2017), and ligaments (Gobbi and Whyte 2017). Compared with the use of scaffolds or ex vivo cultured cell therapies, the clinical use of PRP is of relative practical and/or regulatory convenience as it is autologous, minimally obtainable, and relatively easy to apply. Given the fact that there are numerous PRP usages (such as preparation protocol, formulation, dosage, application technique), there are inconsistencies in the clinical studies as systematically reviewed by Chahla et al. (Chahla et al. 2017). The benefits of PRP depend on the context (Andia and Maffulli 2013), and thus some studies disagree with each other (Metcalf et al. 2013; Piuzzi et al. 2017). Nourissat et al. (Nourissat et al. 2013) pointed out that there is no evidence favouring the use of PRP in arthroscopic surgery while a meta-analysis (Sheth et al. 2012) showed that there was no absolute evidence on the clinical usefulness of PRP in orthopaedics.

## Orthopaedic regenerative tissue engineering in the operating room

### Tendons and ligaments

To meet the extensive clinical needs regarding tendons and ligaments, biomaterials (Table 1) have mainly been used for repair augmentation rather than for direct tissue regeneration since immediate mechanical properties are demanded as the need to overcome the known limitations of grafts. It is not uncommon that the same or similar biomaterials were used for tendons and ligaments. The synthetic materials such as polytetrafluoroethylene (Gore-Tex Device; W.L. Gore, Flagstaff, Arkansas) or Dacron Device (Stryker, Kalamazoo, Michigan) were used in the past (Chen et al. 2015). Polyethylene terephthalate (LARS ligament; LARS, Arc sur Tille, Burgundy, France), and natural polymers such as silk-based biomaterials (SeriACL graft; Serica Technologies, Medford, Massachusetts) as an alternative to grafts (https://clinicaltrials.gov/ct2/show/record/NCT00775892 and https://clinicaltrials.gov/ct2/show/NCT00490594) were tested, all of which having been manufactured with textile technologies to achieve higher mechanical strength. For tendon repair, extracellular matrix and synthetic polymers were envisaged (Derwin et al. 2006; Ricchetti et al. 2012; Smith et al. 2017). Patients that received a polycarbonate polyurethane patch (Biomerix, Fremont, CA) for rotator cuff repair augmentation showed significant improvements in pain relief, simple shoulder test, and American Shoulder and Elbow Surgeons shoulder scores with a 10% re-tear rate at the 12-months post-operation (Encalada-Diaz et al. 2011). The use of Zimmer Collagen Repair Patch in the treatment of extensive rotator cuff tears reported to provide excellent pain relief with a moderate functional improvement (Badhe et al. 2008). In a prospective, randomised controlled trial with GraftJacket Regenerative Tissue Matrix, intact repair ratios were reported to be in 85% in the augmented group and 40% in the control group (Barber et al. 2012). In a case-controlled study (Hernigou et al. 2014), injection of bone marrow concentrates containing mesenchymal stem cells as an adjunct therapy during arthroscopy improved the healing of rotator cuff with tendon integrity found in 87% of patients in the bone marrow concentrate injection group versus 44% in the control group (Hernigou et al. 2014). Based on the randomised controlled trial performed by Ianotti et al. (Iannotti et al. 2006), repair of massive chronic rotator cuff tears was not recommended with Restore Orthobiologic Implant (DePuy Orthopaedics; Warsaw, Indiana) since it did not improve the clinical outcomes or tendon healing with 9 out of 15 cases healed in the control group while only 4 out of 15 cases healing in the augmentation group (Iannotti et al. 2006). Moreover, Zheng et al. (Zheng et al. 2005) observed that patients who received Restore Orthobiologic Implant for tendon repair displayed swelling and severe pain at the implantation site. The study critically analysed the implant through histology and nested polymerase chain reaction. The authors reported the presence of porcine cells and DNA material in contrast to the way it was advertised, and in animal studies, they observed inflammatory responses characterised by massive lymphocyte infiltration (Zheng et al. 2005).

**Table 1:** Examples of commercial products for tendon repair

Product	Company	Biomaterial	References
Ligament Advanced Reinforcement System (LARS)	LARS (Arc sur Tille, France)	Polyethylene terephthalate	(Naim et al. 2011; Gao et al. 2010; Dominkus et al. 2006)
GraftJacket Regenerative Tissue Matrix	LifeCell (Branchburg, New Jersey; distributed by Wright Medical Technology, Arlington, Tennessee)	Processed human dermis	(Wong et al. 2010; Bond et al. 2008; Barber et al. 2012)
Restore Orthobiologic Implant	DePuy Orthopaedics (Warsaw, Indiana)	Collagen-based patch from porcine small intestine submucosa	(Iannotti et al. 2006; Walton et al. 2007; Zheng et al. 2005)
Zimmer Collagen Repair Patch (formerly: Permacol - Tissue Science Laboratories; Aldershot, Hampshire, UK)	Tissue Science Laboratories (Aldershot, Hampshire, United Kingdom; distributed by Zimmer, Warsaw, Indiana)	Processed porcine dermis	(Badhe et al. 2008; Giannotti et al. 2014; Soler et al. 2007)
CuffPatch Bioengineered Tissue Reinforcement	Organogenesis (Canton, Massachusetts; marketed by Arthrotek, Warsaw, Indiana)	Multilayer sheet from porcine small intestine submucosa	(Abraham et al. 2000; Barber et al. 2006; Derwin et al. 2006)
X-Repair	Synthasome (San Diego, California)	Poly-L-lactic acid mesh	(McCarron et al. 2010; Proctor 2014; Smith et al. 2016)
Poly-Tape (Dacron)	Neoligaments (Xiros; Leeds, UK)	Polyethylene terephthalate	(Smith et al. 2016; Smith et al. 2017)
ArthroFlex	Arthrex (Naples, Florida)	Processed human dermis	(Beitzel et al. 2012; Petri et al. 2015)
Bio-Blanket	Stryker Orthopaedics (Mahwah, New Jersey)	Processed bovine dermis	(Chen et al. 2009; Ricchetti et al. 2012; Rotini et al. 2011)
Conexa	Tornier (Edina, Minnesota)	Processed porcine dermis	(Gupta et al. 2013; Shea et al. 2010; Xu et al. 2012)
SportMesh Soft Tissue Reinforcement	Biomet Sports Medicine (Warsaw, Indiana)	Poly(urethaneurea)	(Petriccioli et al. 2013; Barber and Aziz-Jacobo 2009)
TissueMend Soft Tissue Repair Matrix	TEI Biosciences (Boston, Massachusetts; marketed by Stryker Orthopaedics, Mahwah, New Jersey)	Collagen membrane derived from fetal bovine dermis	(Chen et al. 2009; James et al. 2010; Song et al. 2010)

In a case series with a minimum 2-year follow-up of reconstruction of irreparable massive or full-thickness 2-tendon rotator cuff tears using Conexa, significant improvement in pain, range of motion, strength, and subjective outcome measures in patients with minimal glenohumeral arthritis were reported (Gupta et al. 2013). However, this study lacks the long-term results and case-controls. X-Repair (poly-L-lactic acid) was used to reinforce the surgical repair of large to massive rotator cuff tears, and 83% and 78% of patients had important functional improvement at 12 and 48 months post-surgery, respectively (Proctor 2014). The use of a polyethylene terephthalate fibre mesh (Dacron; Dacron Xiros, Leeds, UK) in the surgical augmentation of the symptomatic massive rotator cuff tears provided pain relief and improved shoulder movement with a 90% of mean patient satisfaction score (Nada et al. 2010). However, there were no controls in this study.

### Skeletal muscle

Clinical applications of muscle regeneration strategies are very limited although the topic receives a substantial amount of interest in basic science and preclinical studies (Corona and Greising 2016; Kwee and Mooney 2017). Restore Orthobiologic Implant (DePuy Orthopaedics; Warsaw, Indiana) was also used to treat a patient with large volumetric muscle loss and the presence of new tissue at the implant assessed by computer tomography, showing an improvement in strength after the surgery (Mase et al. 2010). Sicari et al. (Sicari et al. 2014) used extracellular matrix scaffolds from porcine urinary bladder to treat patients with volumetric muscle loss. The scaffolds promoted the remodelling of muscle tissue with perivascular stem cell homing and the de novo formation of muscle cells, and functional improvements in some patients (Sicari et al. 2014).

### Articular cartilage

The current surgical treatments for articular cartilage lesions include microfracture, mosaicplasty, cell implantation, osteochondral allograft transfer arthroscopic chondroplasty, and joint arthroplasty (Mollon et al. 2013). The fast spread of autologous chondrocyte implantation (ACI) (Brittberg et al. 1994; Knutsen et al. 2016) and matrix-induced autologous chondrocyte implantation (MACI) (Basad et al. 2010; Brittberg 2010) indeed promote the growth of the concept of regenerative tissue engineering with numerous papers, although, hyaline-like cartilage is not evidenced by high-quality clinical studies (Mollon et al. 2013).

The Medical Services Advisory Committee in Australia performed an evidence-based assessment in their ACI/MACI report accessible on http://www.msac.gov.au/internet/msac/publishing.nsf/Content/E72BFBEC5447F91FCA25801000123B6D/$File/1140_Report_Final040211.pdf. It was demonstrated that ACI/MACI is safe without serious adverse effects. Nevertheless, the committee reported that ACI/MACI or comparator treatments were not better than non-surgical treatments in high-quality randomised controlled trials. The effectiveness of ACI/MACI was comparable to mosaicplasty and microfracture, i.e. the selected comparator treatments, in terms of function, pain relief, and life quality in short-medium term. The main disadvantages over the comparator treatments are that ACI/MACI requires two surgeries, chondrocytes dedifferentiate during in vitro culture, with costs reported to be of 14 k$ for biopsy and grafting procedure while mosaicplasty costs 2.6 k$ and microfracture 1.4 k$. On the other hand, more recent studies provide evidence favouring MACI over microfacture (Devitt et al. 2017; Saris et al. 2014). In the randomized clinical trial of Saris et al. (Saris et al. 2014), MACI was reported to be clinically significantly better than microfracture in the treatment of symptomatic focal cartilage defects at least 3 cm^2^ in size at a 2-year follow-up, but the structural repair tissue was similar. A systematic review of the randomised controlled trials (Devitt et al. 2017) showed that the defects larger than 4.5 cm^2^ treated with ACI/MACI had better outcomes than with microfracture. Nevertheless, based on the available evidence, no single treatment can be assigned to be the most effective method. This review (Devitt et al. 2017) included studies in which the patients were 18–55 years old with an articular cartilage lesion with a size of 1–15 cm^2^ and an International Cartilage Repair Society grade of II-IV that are not related to osteonecrosis, osteoarthritis, nor inflammatory arthritis.

**Fig. 2:**
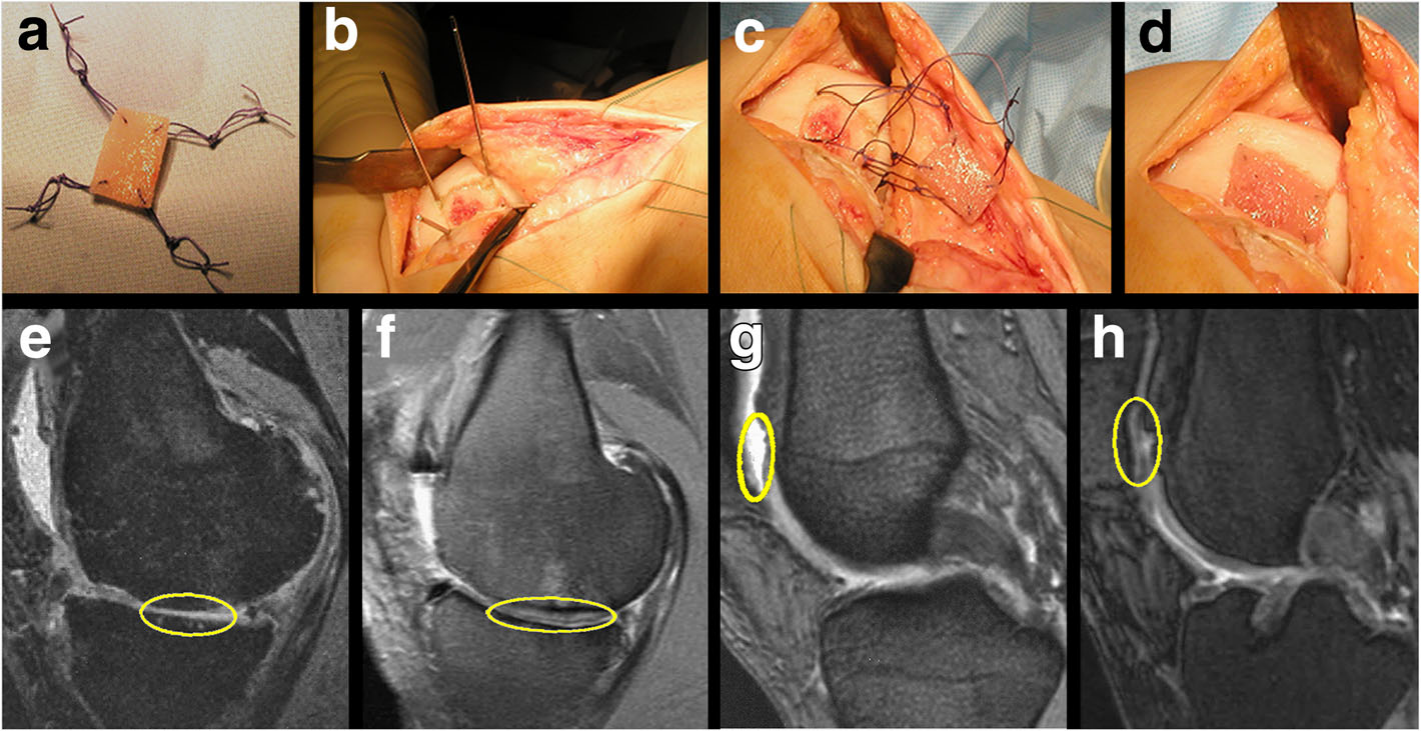
For implantation, BioSeed-C was armed from the corners with resorbable sutures secured by threefold knots (**a**), k-wires were drilled in the corner of the defect (**b**), using the k-wires, the guiding threads were pulled through the femoral bone, and the knots were guided into the subchondral bone (**c**), the knots functioned as anchors, seized the subchondral bone and fixed the implant (**d**), MRI showing the cartilage defect at the medial femoral condyle before implantation (**e**) and after four years where the defect was completely filled (**f**), and the MRI shows a patellar cartilage defect before implantation (**g**) and after four years where defect was completely filled (**h**). Adapted with a minor modification from (Kreuz et al. 2009)

There are many products available (Table 2). The strategies for cartilage regeneration may have a major drawback including the fibrocartilage (and not hyaline) nature of the neo-tissue. Improved strategies are still needed for cartilage and the best cost-effective treatment should be identified with more rigorous prospective, with high quality randomised clinical trials (Vilela et al. 2015). Kreuz et al. (Kreuz et al. 2009) reported the clinical outcome after a 4-year clinical follow-up (Fig. 2) of the focal osteoarthritic knee cartilage defects that were treated with BioSeed-C (BioTissue Technologies GmbH; Freiburg,Germany), a second-generation autologous cartilage graft based on a bioresorbable two-component gel-polymer scaffold. Significant improvement in the Lysholm and International Cartilage Repair Society (ICRS) scores were observed as early as 6 months but remained unchanged during the follow-up. The International Knee Documentation Committee (IKDC) score and Osteoarthritis Outcome Score (KOOS) were also improved. Magnetic resonance imaging (MRI) evaluation showed moderately/completely filled defects while hyper intense signals were seen in 16 out of 19 patients. There was no improvement in the clinical and MRI scores 2 out of 19 patients (Kreuz et al. 2009).

**Table 2:** Examples of commercial products for cartilage repair

Product	Company	Biomaterial + Cells	References
Bioseed-C	BioTissue Technologies (Freiburg, Germany)	Polylactin/polydiaxanon/fibrin + autologous chondrocytes	(Zeifang et al. 2010; Ossendorf et al. 2007; Kreuz et al. 2011)
Chondrosphere (ACT3D-CS/ARTHROCELL 3D)	Co.don (Teltow, Germany)	No scaffold + Autologous chondrocytes	(Becher et al. 2017; Fickert et al. 2012; Siebold et al. 2018)
CaReS-1S	Arthro Kinetics Biotechnology (Krems, Austria)	Murine (rat tail) type-I collagen hydrogel + autologous chondrocytes	(Petri et al. 2013; Schneider et al. 2011)
Biocart II	Histohenics (Waltham, Massachusetts)	Fibrin/hyaluronic acid + autologous chondrocytes	(Eshed et al. 2012; Nehrer et al. 2008)
Cartipatch	Tissue Bank of France (Lyon, France)	Agarose/alginate hydrogel + autologous chondrocytes	(Selmi et al. 2008)
NeoCart	Histogenics (Waltham, Massachusetts)	Bovine type-I collagen + autologous chondrocytes	(Anderson et al. 2017; Crawford et al. 2012; Crawford et al. 2009)
RevaFlex (DeNovo ET)	Isto Technologies (St. Louis, Missouri)	No scaffold + allogeneic juvenile chondrocytes	(McCormick et al. 2013)
Novocart 3D	TETEC Tissue Engineering Technologies (Reutlingen, Germany)	Bovine type-I collagen/chondroitin sulphate + autologous chondrocytes	(Niethammer et al. 2014; Zak et al. 2014; Niethammer et al. 2017)

Gobbi and Whyte (Gobbi and Whyte 2016) evaluated the medium-term clinical outcomes of cartilage repair using a single-stage technique of a hyaluronic acid-based scaffold (Hyalofast; Anika Therapeutics, Abano Terme, Italy) with bone marrow aspirate concentrate compared with microfracture technique. At 2 years post-surgery, good clinical outcomes were obtained with both techniques, while at 5 years post-surgery, patients from the microfracture group could not maintain the improvement unlike patients from the scaffold group (Gobbi and Whyte 2016).

The NeoCart implant is a cartilage scaffold from type-I collagen (Histogenics, Waltham, Massachusetts) designed to be seeded with autologous chondrocytes and cultured in a bioreactor. Preliminary studies showed that it improves the function and pain relief, and works better than microfracture (Crawford et al. 2012). Wakitani et al. (Wakitani et al. 2007) treated cartilage defects with ex vivo cultured autologous bone marrow mesenchymal cell that were placed first embedded in a soluble type-I collagen from bovine skin (Koken, Tokyo, Japan), then placed onto a collagen sheet from porcine tendon (Gunze, Kyoto, Japan), gelated, and further cultured for few days before implantation. Clinical improvements were achieved at 6 months post-surgery, and at 12 months post-surgery the defects were confirmed to be filled while one of the cases’ histology indicated a fibrocartilaginous tissue in the defect, not hyaline cartilage (Wakitani et al. 2007). In a multicenter randomised controlled trial (Shive et al. 2006), a chitosan-glycerol phosphate-based hydrogel, BST-CarGel (Piramal Healthcare Inc., Laval, Quebec, Canada) was reported to be superior at 5 years than microfracture with better tissue repair in quantity and quality while being similar on Western Ontario and McMaster Universities Osteoarthritis Index (WOMAC) tool, showed that there are no clinical differences between the BST-CarGel and microfracture group.

Particulated Articular Cartilage products are also commercially available. Cartilage Autograft Implantation System (CAIS) (DePuy Mitek, Raynham,Massachusetts) is based on the distribution of autogenous cartilage tissue pieces on a polycaprolactone/polyglycolic acid scaffold. With DeNovoNatural Tissue (Zimmer, Warsaw, Indiana), juvenile allograft cartilage pieces are fixed into a defect with fibrin glue (Farr et al. 2012; Mollon et al. 2013). A randomised controlled trial (Cole et al. 2011) compared CAIS and microfracture. CAIS patients had significantly more improvements in the KOOS instrument and IKDC score than the microfracture patients at 24 months post-operation (Cole et al. 2011).

Studies from Hunziker et al. (Hunziker et al. 2015), Iwasa et al. (Iwasa et al. 2009), and Huang et al. (Huang et al. 2016) are of importance as comprehensive reviews on cartilage repair in the clinical situation. Although the biomaterials and strategies differ slightly from each other, none of the products’ outcome is satisfactory enough. It can be concluded that it is not possible to deduct a clear ranking of products, and a firm recommendation on the preferred cartilage repair strategy cannot be made. As already discussed in the previous section, the challenges are many and should be overcome for a robust and novel regenerative treatment of cartilage lesions.

### Bone and osteochondral tissue

Given the composition of natural bone, ceramics alone or combined with polymers found an ample space for applications and provided limited acceptable outcomes thanks to active biology within the bone, such as hydroxyapatite (e.g. Finblock; FinCeramica Faenza, Italy) (Marcacci et al. 2007) and stem cell-loaded β-tricalcium phosphate (β-TCP; Osferion Olympus Biomaterial, Tokyo, Japan) (Kawate et al. 2006).

Oryan et al. comprehensively reviewed the bone grafts and commercially available bone substitutes (which are numerous and thus it is not within the scope of this review to review each of them), and their clinical applications (Oryan et al. 2014). The autografts remain the gold standard for bone regeneration by functioning superior to tissue engineering scaffolds.

Sotome et al. (Sotome et al. 2016) reported the results of a multicenter randomised controlled trial showing that porous hydroxyapatite/collagen composite (Refit; HOYA Technosurgical, Tokyo, Japan) was superior to porous β-tricalcium phosphate implant (Osferion; Olympus, Tokyo, Japan) for bone regeneration but with a relatively higher incidence of adverse (although not serious) effects as no implants were rejected (Sotome et al. 2016). For the treatment of talar osteochondral lesions, two different scaffolds were used to deliver bone marrow-derived cells with a platelet gel. Based on the results of a minimum follow-up of 24 months, both scaffolds (Spongostan Powder: porcine collagen powder, Johnson & Johnson Medical, Gargrave, Skipton, UK; and HYAFF-11: hyaluronic acid, Fidia Advanced Biopolymers Laboratories, Abano Terme, Italy) provided a similar improvement (Giannini et al. 2009).

**Fig. 3:**
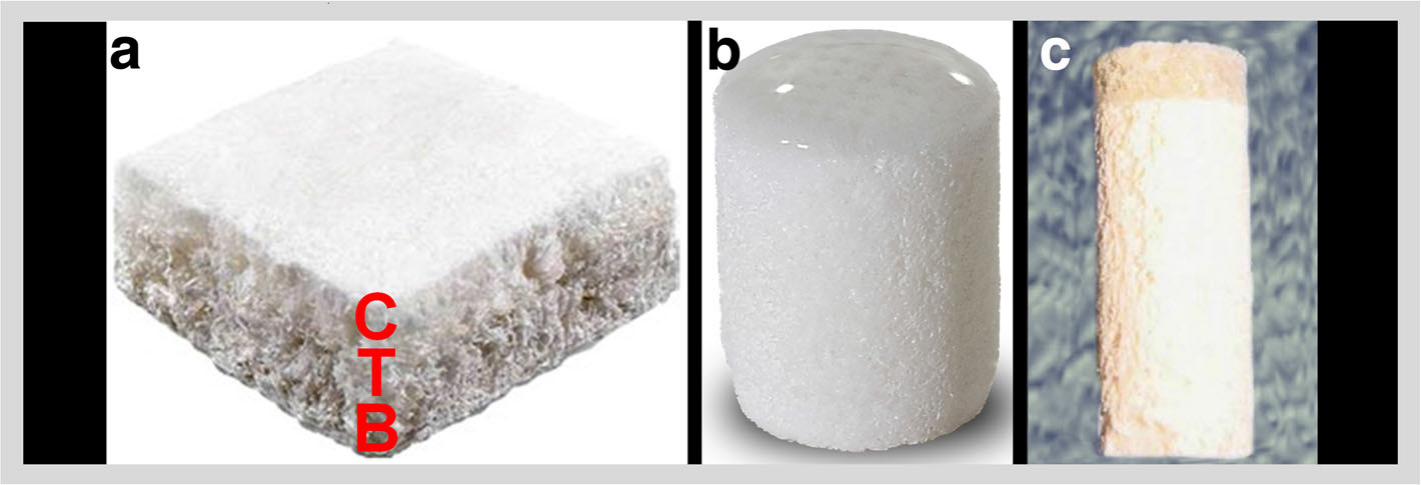
An osteochondral scaffold (MaioRegen, Fin-Ceramica, Faenza, Italy) has a porous 3D tri-layer composite structure to mimic the osteochondral tissue. The top layer (the red C) is for the cartilage tissue and made from type-I collagen, the transition layer (the red T) is for the transition zone, subchondral bone, and is 60% from type-I collagen and 40% from magnesium-hydroxyapatite, and the bottom layer (the red B) is for the bone tissue and is 30% from type-I collagen and 70% from magnesium-hydroxyapatite (**a**), a bilayer osteochondral scaffold (Agili-C; CartiHeal, Israel) that is currently in clinical trial and made from aragonite and hyaluronic acid shape of cylinders, with a similar surgical technique as the mosaic-like osteochondral transplantation. The top layer is for the cartilage tissue and is from aragonite and hyaluronic acid, and the bottom layer is for bone tissue phase and is from calcium carbonate in the aragonite crystalline form (**b**), a bilayer scaffold (TruFit CB; Smith & Nephew, Andover, Massachusetts) is from poly(lactic-co-glycolic acid), poly(glycolic acid) fibres, and calcium sulphate (**c**). Adapted with a minor modification from (Kon et al. 2014)

Composites and multilayer scaffolds are of interest for osteochondral tissue engineering where each layer is designed for a particular tissue (Fig. 3). In the pilot clinical trial of MaioRegen (Fin-Ceramica, Faenza, Italy) for the treatment of 15 osteochondral defects (Fin-Ceramica, Faenza, Italy), Kon et al. (Kon et al. 2010) reported promising preliminary clinical results at short-term are reported. In 13 defects, the scaffolds were completely attached and a repair tissue was present, in 10 defects the defects were completely filled at 6 months post-implantation, and complete integration almost only in half of the defects (in 8 defects). At 6 months post-implantation, significant improvements in the IKDC scores were reported although oedema or sclerosis in the subchondral bone were found in 5 defects (Kon et al. 2010). Altered MRI signal and a slow maturation process were also reported (Kon et al. 2014). With The TruFit CB scaffold (Smith & Nephew, Andover, Massachusetts) (Melton et al. 2010), controversial clinical and imaging outcomes were reported (Kon et al. 2014) and its commercialisation suspended (Vilela et al. 2015).

### Meniscus

**Fig. 4:**
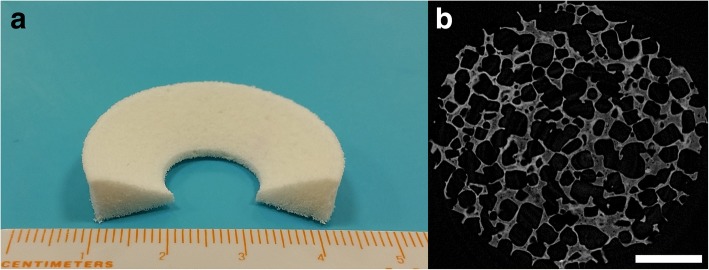
A photograph (**a**) and micro-structural image (**b**) of a commercially available polycaprolactone-polyurethane scaffold (Actifit; Orteq Bioengineering, London, UK) for meniscus. The scale bar indicates 1 mm

The clinical management of meniscus lesions is sometimes challenging, with the primary intention to preserve the meniscus as much as possible and repairing has provided good results in some cases; indications for scaffolds are limited (Cengiz et al. 2017b; Cengiz et al. 2017d; Pereira et al. 2016a; Pereira et al. 2016b). Two scaffolds are available: i) bovine type-I collagen scaffold (CMI; Ivy Sports Medicine, Lochhamer, Germany) (Monllau et al. 2011; Zaffagnini et al. 2015; Zaffagnini et al. 2011), and ii) polycaprolactone-polyurethane scaffold (Actifit; Orteq Bioengineering, London, UK) (Fig. 4) (Bouyarmane et al. 2014; Bulgheroni et al. 2013; Gelber et al. 2015; Verdonk et al. 2012). The commercial scaffolds are safe without apparent adverse reactions and somewhat positive functional and clinical results have been achieved despite their limitations. However, when native-like meniscus regeneration is questioned, there are some outstanding issues to be addressed (matrix composition and organisation of the neo-tissue). Nevertheless, the restoration of the fundamental function of meniscus, i.e. the protection of cartilage, should be one of the primary goals since obtaining a functionless repair tissue will not help the patient in the long-term (Cengiz et al. 2017b).

Due to their regenerative potential and regulatory and practical advantage (Chahla et al. 2017), biologics including platelet-rich plasma (PRP), bone marrow aspirate concentrate, hyaluronic acid, and stem cells (Gobbi et al. 2017; Narayanan et al. 2017) have been widely used in orthopaedics (Gobbi et al. 2017; Musahl et al. 2017; Utku et al. 2015) including for bone (Malhotra et al. 2013), cartilage (Nakasa et al. 2017), meniscus (Cengiz et al. 2017a), muscle (Canata et al. 2017), tendons (Canata et al. 2017), and ligaments (Gobbi and Whyte 2017). Compared with the use of scaffolds or ex vivo cultured cell therapies, the clinical use of PRP is of relative practical and/or regulatory convenience as it is autologous, minimally obtainable, and relatively easy to apply. Given the fact that there are numerous PRP usages (such as preparation protocol, formulation, dosage, application technique), there are inconsistencies in the clinical studies as systematically reviewed by Chahla et al. (Chahla et al. 2017). The benefits of PRP depend on the context (Andia and Maffulli 2013), and thus some studies disagree with each other (Metcalf et al. 2013; Piuzzi et al. 2017). Nourissat et al. (Nourissat et al. 2013) pointed out that there is no evidence favouring the use of PRP in arthroscopic surgery while a meta-analysis (Sheth et al. 2012) showed that there was no absolute evidence on the clinical usefulness of PRP in orthopaedics.

## Conclusions and final remarks

There have been several exciting advances in orthopaedic tissue engineering. Many lessons have been learned. Some issues were already solved, but many still need to be addressed while future problems may also arise. Reaching and getting through a clinical trial is a time-consuming, laborious, and expensive path, while all these things get exponential once entering the operating room. Based on the current evidence, there is a disequilibrium between the demand and supply in the clinics, and tissue engineering has still ample room to grow before delivering fully functional solutions. This mandatory growth will be an outcome of the teamwork between the scientific researchers, orthopaedic surgeons, research funding bodies, industry, investors, governmental bodies, and regulatory bodies. Orthopaedic tissues are 3D solid/filled tissues that perform mechanically, thus scaffolds are and will be an indispensable volume filling and mechanically supporting component for large lesions. Future scaffold-based regeneration strategies should be evidenced to have: (i) superior tissue regeneration capability with excellent long-term clinical outcomes, and (ii) comparable cost with the existing methods. For this, the challenges should be recognised and addressed. Systematic reviews and meta-analyses are worthy and bring original results from existing results. Although it may not be so easy, a superior research and funding mind-set seems to be necessary to encourage academic scientists and clinicians to execute studies that seek long-term outcomes, and high quality randomised clinical trials that take many years and consume a tremendous amount of money but yield only one paper eventually. With its dynamic and enthusiastic nature, tissue engineering may hopefully enable a major change in the clinical management of orthopaedic disorders in the future, and in particular to personalised approaches, with only one ultimate goal – the healthier individuals.

## Abbreviations

ACI: Autologous chondrocyte implantation
ICRS: International Cartilage Repair Society
IKDC: International Knee Documentation Committee
KOOS: Osteoarthritis Outcome Score
MACI: Matrix-induced autologous chondrocyte implantation
MRI: Magnetic resonance image
PRP: Platelet-rich plasma
WOMAC: Western Ontario and McMaster Universities Osteoarthritis Index
